# Synthesis of Metal–Saccharin Complexes as an Antimicrobial Inorganic Pigment for Surfaces

**DOI:** 10.1021/acsomega.5c07778

**Published:** 2025-11-12

**Authors:** Camila Acorone Soares, Patrícia Appelt, Weslei Domingos da Silva, Mário Antônio Alves da Cunha, Tulio Chavez-Gil, Henrique E. Toma, Davi F. Back, Fauze Jacó Anaissi

**Affiliations:** † Department of Chemistry, Campus Cedeteg, 307046 Universidade Estadual do Centro-Oeste, Guarapuava 85040-080, Brazil; ‡ Department of Chemistry, Campus Pato Branco, Universidade Tecnológica Federal do Paraná, Pato Branco 85503-390, Brazil; § Laboratory of Advanced Biofuels and Biomaterials, Department of Natural Sciences, 1473 Coppin State University, 2500 W North Avenue, Baltimore, Maryland 21216, United States; ∥ Department of Chemistry, University of São Paulo (USP), Avenue Professor Lineu Prestes, São Paulo, São Paulo 05508-000, Brazil; ⊥ Department of Chemistry, Universidade Tecnológica Federal de Santa Maria (UFSM), Avenue Roraima 1000, Santa Maria, Rio Grande do Sul 97105-900, Brazil

## Abstract

There is significant concern within the scientific community regarding the prevention and treatment of diseases caused by viruses and bacteria as well as the increasing resistance of these microorganisms to conventional medicines. Numerous studies have addressed this challenge to explore the antimicrobial potential of various compounds and improve existing drugs, aiming to counteract microbial mutations and prevent large-scale outbreaks. This study synthesized and characterized three metal–saccharin complexes (Fe, Co, and Ni) and investigated their application as antimicrobial activity pigments. The complexes were studied using molar conductivity, Fourier transform infrared spectroscopy, mass spectrometry, nuclear magnetic resonance (NMR ^1^H), UV–vis spectroscopy, thermogravimetry, single-crystal X-ray diffraction, and colorimetry. The results confirmed the formation and composition of the metal complexes. The synthesized compounds exhibited antimicrobial activity in both solution and solid states, maintaining their efficacy when incorporated into acrylic paste and fabric paint. The Co–sac complex displayed superior antimicrobial activity among the tested complexes across all evaluations. It inhibited the growth of *Salmonella typhimurium* and *Candida albicans* at a MIC of 62.5 μg·mL^–1^, with fungicidal and bactericidal effects confirmed at 125 μg·mL^–1^. In disk diffusion assays, Co–sac (10%) in acrylic paste showed a maximum inhibition halo of 14.03 ± 0.39 mm against *Staphylococcus aureus*. When applied to face mask fabric, the same complex achieved inhibition halos of up to 11.65 ± 2.25 mm for *Escherichia coli* and 8.78 ± 2.34 mm for *Listeria monocytogenes*. Notably, the antimicrobial activity index reached values as high as 1.05, surpassing those of conventional antibiotics in specific tests.

## Graphical abstract

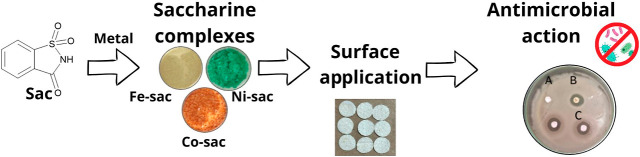

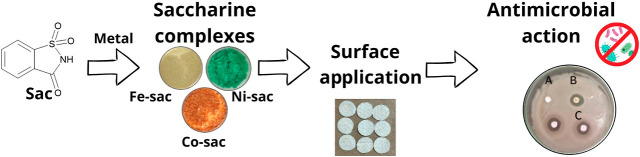

## 1 Introduction

Currently, it is estimated that each year, about 4.95 million people die from infections caused by antimicrobial resistance, with 1.27 million deaths directly attributable to drug-resistant bacterial infections.^1,2^ This situation has primarily arisen from the extensive use of drugs as well as the indiscriminate use by the patient himself.^3,4^ The overuse and misuse of antibiotics have accelerated the evolution and dissemination of resistant strains, undermining the effectiveness of standard treatments and posing a serious threat to global health.^5^

Preventing infections caused by viruses and bacteria, as well as the resistance these microorganisms develop to medications, has been a growing concern in society.^6^ The increase in resistance decreases the effectiveness of drug treatment and makes the disease difficult to treat, increasing the spread of these pathogens.^7^ For this reason, studies should be carried out to improve existing drugs and synthesize new drugs, aiming to reduce the spread of these pathogens and address mutations that hinder effective treatment.^8–10^

Antimicrobials are therapeutic agents that prevent or treat infections, including antibiotics,^11,12^ antivirals,^13,14^ and antifungals.^15,16^ These substances kill microorganisms or inhibit their growth, aiming to maintain essential processes in cellular metabolism, such as the synthesis of biological macromolecules, enzymatic activity, and the integrity of cellular structures such as the cell wall and membranes.^17,18^

*Ortho*-sulfobenzimide (C_7_H_4_NSO_3_), commonly known as saccharin, is a non-nutritive artificial sweetener widely used in the pharmaceutical industry.^19^ It is incorporated as a supplement into various medicinal products, including syrups and suspensions.^20^ Its broad chemical application is attributed to its chelating properties resulting from its polyfunctional structure.^21^ The molecule contains an amino group and can easily be converted into the saccharin anion as a versatile ligand for coordination and yield metal complexes. In its anionic form, it can easily coordinate with metal centers in multiple ways, such as monodentate, bidentate, or tridentate ligand due to the availability of deprotonated nitrogen, carbonyl oxygen, as well as two sulfonic oxygens.^22^

Given these versatile coordination modes, there has been a growing interest in the biological evaluation of metal complexes containing saccharin ligands over the last two decades (2004–2024). Although significant progress has been made in this field, no comprehensive reviews on the anticancer and antimicrobial activities of saccharin or thiosaccharin-based metal complexes are currently available in the literature.^23^

However, recent studies have highlighted the potential of saccharin-derived metal complexes in various applications,^24^ including catalysis, carbonic anhydrase inhibition, controlled drug release, and their use as therapeutic additives in dentifrices.^25,26^

Beyond these applications, incorporating saccharinate copper­(II) (Cu–sac) into paint has shown promising results as an antimicrobial pigment for surface coatings.^26^ The antimicrobial activity of Cu–sac was tested in solution as pellets and incorporated into paint at two concentrations. The tests were performed using the disk diffusion method against ten microbial strains. The complex exhibited significant antimicrobial activity in all forms tested. The activity index, calculated based on the inhibition zones, demonstrated favorable results, particularly for *S. epidermidis* and *Escherichia faecium*, with indices equal to or greater than 1.

Furthermore, incorporating antimicrobial compounds into surfaces, textiles, paints, and the functionalization of materials has become a promising approach to controlling the spread of pathogens in environments with high contamination risk.^27–29^ In this context, the functionalization of textiles with antimicrobial agents, such as metallic nanoparticles, quaternary ammonium compounds, and dyes, with photodynamic properties has shown remarkable efficacy. A particularly innovative approach employed by da Silva et al.^28^ involves using amino-functionalized mesoporous silica nanoparticles as nanocarriers for photosensitizers, enabling the generation of reactive oxygen species upon light activation. These functionalized fabrics exhibit potent antibacterial effects against *Escherichia coli* and *Staphylococcus aureus*.

Therefore, there has been growing interest in synthesizing and characterizing metal–saccharin complexes, as they can form a wide range of coordination compounds with diverse geometries and oxidation states while exhibiting potential biological and pharmacological effects. In this study, we investigated metal–saccharin complexes. We evaluated their ability to inhibit the growth of bacteria and fungi, with the aim of future applications in surface coatings or paints.^30^

## 2 Materials and Methods

### 2.1 Materials

Reagents with a degree of purity appropriate for the experimental tests were used, such as iron­(II) sulfate heptahydrate (FeSO_4_·7H_2_O; Synth, Brazil), cobalt­(II) chloride hexahydrate (CoCl_2_·6H_2_O; NEON, Brazil), nickel­(II) chloride hexahydrate (NEON, Brazil), saccharin sodium dihydrate (C_7_H_4_NO_3_SNa·2H_2_O; Dinâmica, Brazil); methanol (CH_3_OH; P.A.; Synthec, Brazil), ethanol (C_2_H_5_OH; P.A.; Synthec, Brazil), dimethyl sulfoxide DMSO (Biotec, Brazil), and Ultra-Pure (Type 1) Water (Direct Q-3, 18.2 MΩ ατ 25 °C). The following reagents were used: Sabouraud dextrose agar, Miller–Hilton agar (Mueller Hinton Agar) and broth (Mueller Hinton Broth) (both from Sigma-Aldrich, USA), white paint base (Anjo Tintas, Brazil), acrylic paste (AP) (Acrilex, Brazil), respiratory mask (Descarpack, Brazil), tetracycline (30 μg-Laborclin, Brazil), norfloxacin (10 μg-Laborclin, Brazil), and resazurin (Sigma-Aldrich) for biological tests.

### 2.2 Synthesis of the Complexes

The syntheses of the complexes were performed by a similar route for all metals. Saccharin (100 mg, 0.40 mmol) was added to a beaker containing the precursor metal salt in 60 mL of ultrapure water, and the mixture was stirred. The solution was then mixed and heated at 70 °C for 1 h. It was then cooled to room temperature, placed in an ice bath, and allowed to stand for crystallization. The solid was filtered, washed with cold water, dried, and stored for use. Elemental chemical analysis (CHN) shows the formation of complexes with stoichiometric differences. The calculated and found data are summarized below. Suitable single crystals were obtained by slow evaporation of methanol/ethyl ether.

Fe–sac—[Fe­(sac)_2_(H_2_O)_4_]·H_2_O—[tetraaquabis­(1,1,3-trioxo-3*H*-1,2-benzoisothiazol-2-yl)-iron­(II)]­hydrate Iron­(II) sulfate heptahydrate (55 mg, 0.20 mmol), and Na–saccharin (sac) (100 mg, 0.40 mmol). Yield: 98 mg −80%. UV–vis (CH_3_OH): l/nm (ε/L mol^1^ cm^1^) 268 (1.8 × 10^2^), 511 (1.2 × 10^3^).

Co–sac—[Co­(sac)_2_(H_2_O)_4_]·H_2_O—[tetraaquabis­(1,1,3-trioxo-3*H*-1,2-benzoisothiazol-2-yl)-cobalt­(II)]­hydrate cobalt­(II) chloride hexahydrate (48 mg, 0.20 mmol) and Na–saccharin (sac) (100 mg, 0.40 mmol). Yield: 105 mg −86%. UV–vis (CH_3_OH): λ/nm (ε/L mol^1^ cm^1^) 268 (2.7 × 10^2^).

Ni–sac—[Ni­(sac)_2_(H_2_O)_4_]·H_2_O—[tetraaquabis­(1,1,3-trioxo-3*H*-1,2-benzoisothiazol-2-yl)-nickel­(II)]­hydrate nickel­(II) chloride hexahydrate (48 mg, 0.20 mmol) and sodium saccharin (sac) (100 mg, 0.40 mmol). Yield: 104 mg −89%. UV–vis (CH_3_OH): l/nm (ε/L mol^1^ cm^1^) 268 (2.7 × 10^2^), 400 (0.7 × 10^2^), 668 (2.7 × 10^2^), 735 (2.8 × 10^2^).

### 2.3 Characterization Techniques

Infrared spectra of the solid samples were recorded on a PerkinElmer spectrophotometer (FTIR) in the region of 4000–650 cm^–1^. UV–vis spectra in solution were recorded on a Shimadzu spectrophotometer UV-1800, in the range of 190–900 nm, using a quartz tube with a 1 cm optical path length. For the analysis, stock solutions were prepared in methanol at 0.1 mol L^–1^ concentration, while for Co–Sac and Ni–Sac, their concentration was 1.0 × 10^–3^ mol L^–1^, which is also used for Na–sac and Fe–Sac complexes. The molar absorption coefficient (ε) values were calculated according to the Lambert–Beer law, utilizing several consecutive absorbance measurements at a determinate wavelength for solutions with different concentrations. Electronic UV–vis spectra on solid samples were recorded on an Ocean Optics system, composed of a tungsten halogen lamp LS-1 3100K, and in a dark room. The thermogravimetric analyses were performed on a PerkinElmer STA 6000 with automatic DSC, using temperatures ranging from 30 to 900 °C under an air flow atmosphere. Colorimetric analysis was carried out in a portable digital colorimeter, NR145 from 3NH, with an 8 mm measurement opening lid and a light source, a D65 lamp.

Mass spectra (MS) were obtained from a solution of methanol injected into a MicroToF Bruker Daltonics equipment, TOF (MS), with high resolution, in positive ion and ionization by electrospray mode. Nitrogen was used as the nebulizing gas at a pressure of 12 psi and a potential of 4500 V potential. A flow rate of 4 L min^–1^ was maintained at 180 °C. The NMR experiments were conducted at 298 K on an AVANCE-III system with a sample detected by an Ascend-400 MHz Bruker Spectrometer; data analysis was performed with Mnova software. The spectrometer, operating at 9.4 T, is equipped with a 5 mm quadrupolar nuclei direct detection probe for ^1^H; all the NMR chemical shifts are given in ppm relative to an internal standard TMS. The saccharinate complexes were solubilized in deuterated acetic acid *d*_4_ (CD_3_COOD; ≥99% purity).

Single-crystal X-ray diffraction (SCXRD) data were collected on a Bruker APEX II CCD diffractometer equipped with a graphite-monochromatic Mo Kα radiation source (λ = 0.7107 Å). Structure solution and refinement were performed using SHELXL software. The positions of non-hydrogen atoms were located from Fourier difference maps. All non-hydrogen atoms were refined anisotropically by full-matrix least-squares methods based on *F* ^2^. Hydrogen atoms were placed in calculated positions and included in the refinement by using a riding model.

### 2.4 Assessment of the Antimicrobial Potential

The antimicrobial capacity of metal–saccharin complexes, acrylic paste (AP), and white paint incorporated with complexes was studied. Disc diffusion and broth microdilution tests were used to evaluate the degree of microbial susceptibility to the samples. Antimicrobial testing followed protocols described by the Clinical and Laboratory Standards Institute (CLSI).^31,32^

The disk diffusion test was employed to evaluate the antimicrobial potential of acrylic paste infused with metal–complex (Co–Sac, Ni–Sac) samples against *Salmonella enterica* Typhimurium (ATCC 0028) (Gram-negative) and *S. aureus* (ATCC 25923) (Gram-positive). The same test was conducted on respiratory mask (RM) samples painted with fabric paint containing complexes in varying concentrations. In this assay, the following strains were tested: *S. aureus* (ATCC 25923), *Listeria monocytogenes* (ATCC 19111) (Gram-positive), and *E. coli* (ATCC 25922) (Gram-negative). Tetracycline (Tcy) and norfloxacin (Nor) were utilized as antibiotic controls.

Samples of unpainted respirator masks and pure acrylic paste were used as negative controls. The antibacterial experiments were conducted (in duplicate) by impregnating commercial disks of 6 mm diameter with metal–saccharine solutions and placing them systematically in Petri dishes on the culture media (Mueller–Hinton Agar) and incubating at 37 °C for 24 h. After incubation, the inhibition zones were measured with a digital caliper. The antibacterial effectiveness of M–sac complexes was evaluated at concentrations of 5% and 10% for each complex. The antibacterial activity index (AI) and percentage activity (PI) were calculated according to Schons et al. (2023).^33^

The minimum inhibitory concentrations (MIC) of Fe–Sac, Co–Sac, and Ni–Sac samples and their biocidal and biostatic effects were evaluated against the Gram-negative *S. enterica serovar Typhimurium* (ATCC 0028) and the yeast *Candida albicans* (ATCC 10231), respectively. The inoculum concentration was adjusted at the 0.5 McFarland scale (1.5 × 10^8^ cfu mL^–1^) using a UV–vis spectrophotometer on the 625 nm wavelength. Samples (1000–62.5 μg μL-1) were dispersed in 6.25% dimethyl sulfoxide (DMSO); chloramphenicol was used as a positive control for the bacteria and fluconazole for the yeast (*C. albicans*). The solvent DMSO at a concentration of 6.25% was used as the negative control to confirm that the solvent does not have antimicrobial activity in this concentration. The plate was incubated at 37 °C for 24 h for bacterial growth and at 28 °C for 48 h for fungal cultivation. After the incubation, the indicator resazurin (1 mg mL^–1^) was added to all wells, and the plates were incubated for two additional hours. The wells that exhibited microbial inhibition were tested to determine whether the compounds had bactericidal or bacteriostatic activity (using the minimum bactericidal concentration or MBC method) or fungicidal or fungistatic activity (using the minimum fungicidal concentration or MFC method).^35^ At this stage, microbial growth indicates that the compound has only bacteriostatic activity, while microbial death confirms bactericidal action.^34^

## 3 Results and Discussion

### 3.1 Synthesis

The saccharin ligand reacts with different metal ions to form [M­(sac)_2_(H_2_O)_4_]­H_2_O (sac = saccharin). All complexes are air-stable, have solubilities in various solvents (Table 1), and are obtained in high yields (over 70%). The Fe–sac complex yielded a yellow color; the Co–sac complex yielded a salmon color; and the Ni–sac complex yielded a green color (Scheme 1).

**Table 1:** Results of Solubility and Molar Conductivity of the M–Sac Complexes^a^

solvent	Fe–sac	Co–sac	Ni–sac
water	insoluble	insoluble	insoluble
methanol	soluble	soluble	soluble
ethanol	soluble	soluble	soluble
acetonitrile	soluble	soluble	soluble
dimethyl sulfoxide	soluble	soluble	soluble
tetrahydrofuran	partially soluble	soluble	partially soluble
molar conductivity (Λ)	27.11	27.21	26.07

a Molar conductivity (μS cm^1^) of 10^–3^ M solutions in methanol.

**Scheme 1:**
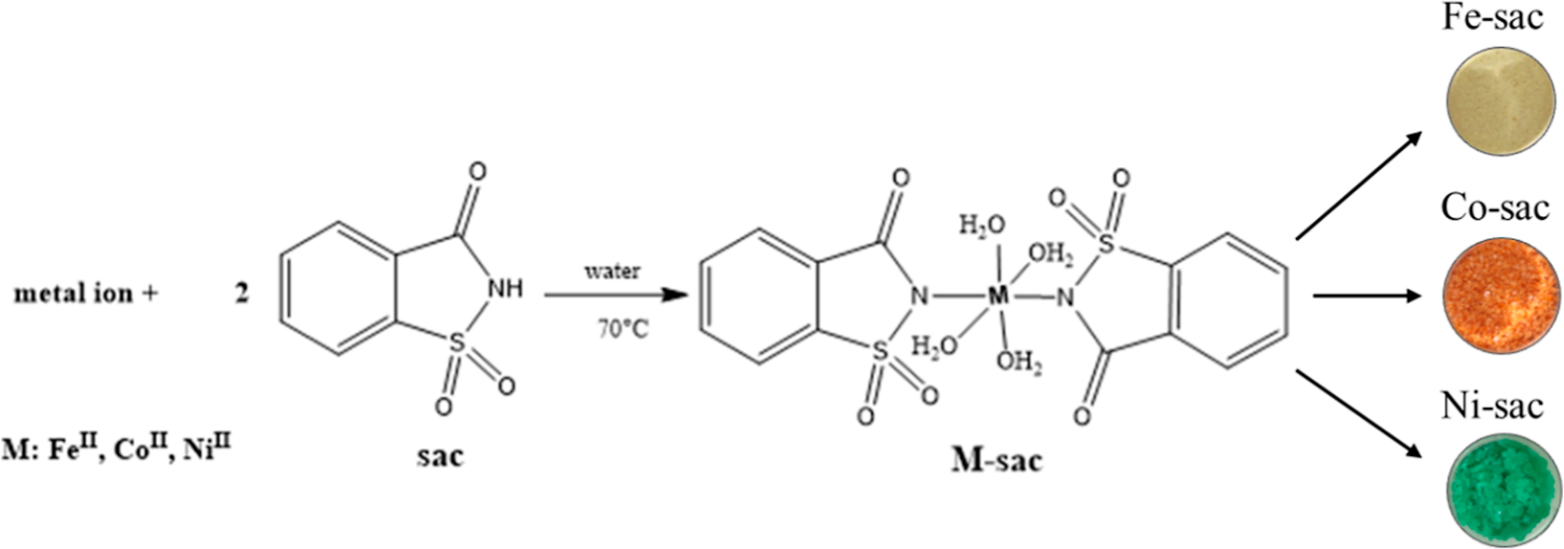
Synthesis of Complexes of M–Sac

### 3.2 Solubility and Conductivity

Table 2 shows the synthesized complexes’ solubility and molar conductivity (in methanol). The saccharinate complexes presented hydrophobic characteristics; therefore, they were soluble only in organic solvents. The molar conductivity of the M–sac complexes (methanol, 1 mmol L^–1^) is very similar; the values of all complexes are compatible with nonconducting solutions, supporting the formation of neutral complexes.^35^

**Table 2:** Thermal Peaks Observed in TG and DTG Curves of Complexes Containing Saccharin

			mass loss (%)		
step	temperature (°C)	dTG peak (°C)	experimental	theoretical	attribution
Na–Sac					
1	30–160	119	5.62	5.60	0.75 mol H_2_O
2	400–550	473	29.56	26.56	1 mol SO_2_
3	550–900	733	35.10	34.86	1 mol C_7_H_4_N
residue	900		29.71	25.70	Na_2_O
Fe–Sac					
1	30–200	133	18.50	18.25	5 mol H_2_O
2	200–400	368	18.99	25.96	2 mol SO_2_
3	460–900	419	27.62	41.40	2 mol C_7_H_4_N
residue	900		34.89	35.59	Fe_2_O_3_
Co–Sac					
1	30–205	124	20.36	21.80	6 mol H_2_O
2	370–460	416	22.45	25.86	2 mol SO_2_
3	460–900	523	35.55	41.23	2 mol C_7_H_4_N
residue	900		21.64	11.10	Co_3_O_4_
Ni–Sac					
1	30–180	128	18.12	18.10	5 mol H_2_O
2	380–880	421	62.83	66.86	2 mol C_7_H_4_NSO_2_
residue	880		19.05	15.04	NiO

### 3.3 Thermogravimetric Analysis (TG/dTG)

The mass loss curves obtained by thermogravimetric analysis are shown in Figure S1. Sodium saccharin showed three stages of mass loss (Table 2). The first stage occurred between 30 and 160 °C, related to the dehydration of the compound, with the loss of 0.75 mol of H_2_O. The second event occurred between 400 and 550 °C and corresponds to the loss of a SO_2_ molecule. The third stage occurred between 550 and 900 °C and agrees with the decomposition of the organic part of the molecule. Finally, the residue is sodium oxide.

**Figure 1:**
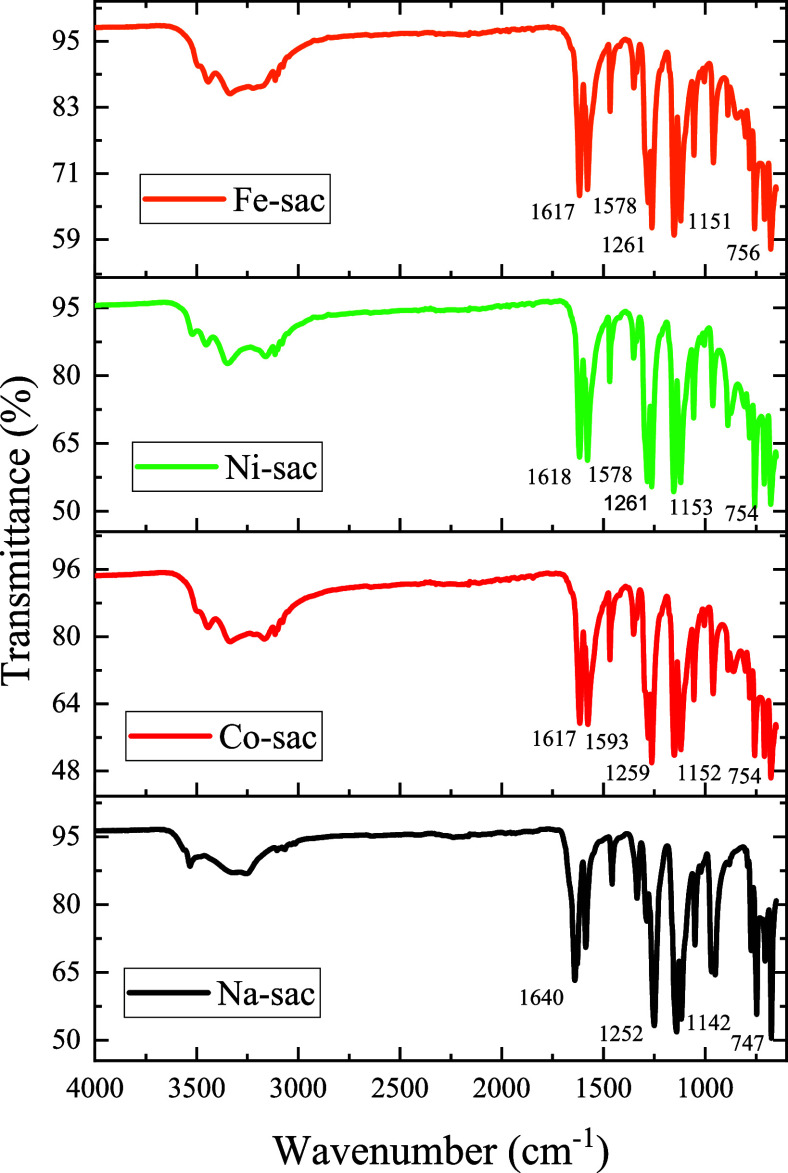
FTIR spectrum of metal–saccharine complexes ATR mode.

The Fe–sac complex decomposed into three steps (Table 2). The first stage, between 30 and 200 °C, is related to the compound’s dehydration, losing five mol of H_2_O. The second stage, between 200 and 400 °C, corresponds to the loss of two SO_2_ molecules. The third stage occurred between 400 and 800 °C and corresponds to the decomposition of the organic matter in the molecule, forming iron oxide III as a residue.

The Co–sac complex decomposed into three steps with a distribution similar to Fe–sac (Figure S1). The first stage is related to the loss of six mol of H_2_O. The second corresponds to the loss of two SO_2_ molecules. The third stage involves the decomposition of organic matter in the molecule, forming cobalt oxide (II and II) as residues (Table 2).

The Ni–sac complex decomposed into two stages. The first stage occurred between 30 and 180 °C and is related to the compound’s dehydration, with the loss of five mol of H_2_O. The second, between 370 and 460 °C, corresponds to the decomposition of the molecule’s saccharin ligands, forming nickel oxide as a residue (Table 2).

### 3.4 FTIR and NMR

Figure 1 shows the Fourier Transform Infrared (FTIR) spectra for the obtained complexes and the free ligand in the ATR method. The data collected allow us to characterize the side chain stretching attributed to functional groups corresponding to the saccharin ligand; and stretching that indicates the chemical coordination of saccharin with the metal centers.

According to Baran and Yilmaz (2006), the FTIR spectra of saccharinates are divided into three regions.^20^ The first region, with bands at approximately 3080_sy_ and 3020_assy_ cm^–1^, correspond to the asymmetric and symmetrical vibration of C–H, respectively.^36^

The second region, where saccharinates have intense bands, is around 1642 cm^–1^, corresponding to the stretching of the carbonyl C=O group, which makes up one of the most critical regions. In the present work, the spectrum of the saccharin molecule alone shows only one strong band corresponding to the carbonyl group at 1725 cm^–1^, while in the complexes formed, two strong bands appear at approximately 1620 and 1570 cm^–1^, making the Co–sac and Fe–sac stretching bands more bathochromically shifted, indicating a strong coordination between the saccharin moiety and the metal ion. In the same region, the aromatic C=C stretching is assigned to elongation that occurs approximately between 1600 and 1462 cm^–1^.^37^

In addition, saccharin, when coordinated in a complex, has another significant region where strong bands depict characteristics of symmetry/asymmetry stretching, which are ascribed to the sulfonite (SO_2_) group that appears at 1280 and 1150 cm^–1^, respectively, and that were observed in all of the synthesized complexes.^38^

For free saccharin, these bands are found at 1330 and 1150 cm^–1^, so this region also undergoes a stretching shift of approximately 50 cm^–1^ once saccharin is part of the chemical coordination. Jovanovski et al. also observed this behavior when they obtained complexes of the type Na_3_(C_7_H_4_NSO_3_)_9_·2H_2_O.^39^

Also, in complexes of [Co­(sac)_2_(mpy)_2_] and [Ni­(sac)_2_(mpy)_2_] (mpy ligand: pyridine-2-methanol) reported by Yilmaz et al.,^40^ these vibrations have a moderate shift of about 25 cm^–1^. Finally, in the region of 1115, 1050, and 1000 cm^–1^, strong bands are observed and are attributed to bending stretches on the equatorial plane for the C–H bonding in the aromatic ring, and a stretch at 755 cm^–1^ is attributed to an out-of-plane flexion of the C–H bond on the aromatic ring.^37^

The ^1^H NMR spectra of the complexes and the free ligand are provided in the Supporting Information. The Supporting Information provides the NMR ^1^H spectra of the complexes and the ligand. The ^1^H NMR spectrum of free saccharin shows the aromatic hydrogens, with a set of doublets and a multiplet in the region of 7.86–8.05 ppm (Figure S3.1), consistent with the literature.^41^ The ^13^C NMR spectrum of free saccharin displays the seven carbons as expected, including the characteristic carbonyl carbon at ∼162 ppm and signals corresponding to the aromatic carbons between 120 and 140 ppm (Figure S3.2).^42^

In the complexes’ ^1^H NMR spectra, the proton resonances are less well resolved than those of the free ligand, a feature of metal coordination. Nonetheless, the aromatic proton signals of saccharin are observed in all spectra, exhibiting slight variations in chemical shift.^43^

For the Fe–sac complex, multiplets are observed in the 7.89–8.07 ppm region (Figure S3.3). The aromatic proton region exhibits characteristic signals for the Co–sac and Ni–sac complexes (Figures S3.4 and S3.5), but they appear as broad and poorly resolved peaks. This behavior is consistent with previous reports, complexes of the general formula M­(sac)_2_·6H_2_O, where M = Fe, Co, Ni, or Mn, present paramagnetic character in solution due to the presence of unpaired d electrons, which hinders the clarity of well-defined ^1^H signals, thereby preventing the observation of sharp ^1^H signals.^43,44^ Consequently, while solution ^1^H NMR data provide structural information, solid-state techniques (vide Section 3.7) enable reliable confirmation of the proposed coordination environment, which aligns with the literature precedent.

### 3.5 Mass Spectrum

The mass spectra were obtained from the complexes previously dissolved in solution. Due to the metal ions’ intrinsic lability, the spray dryer process can promote dehydration or recombination of the ions and ligands, resulting in a broad distribution of complexes.

Figure 2 shows the mass spectroscopy of the Fe–sac sample corresponding to the *m*/*z* peaks. The prominent peak at *m*/*z* 657 corresponds to the theoretical molecular mass of the formation of the Fe–sac complex with the three saccharin ligands [Fe­(C_7_H_4_SO_3_N)_3_]·3­(H_2_O); the peak at *m*/*z* 520 corresponds to the disaccharinate form [Fe­(C_7_H_4_SO_3_N)_2_(H_2_O)_2_]·2­(CH_4_O).

**Figure 2:**
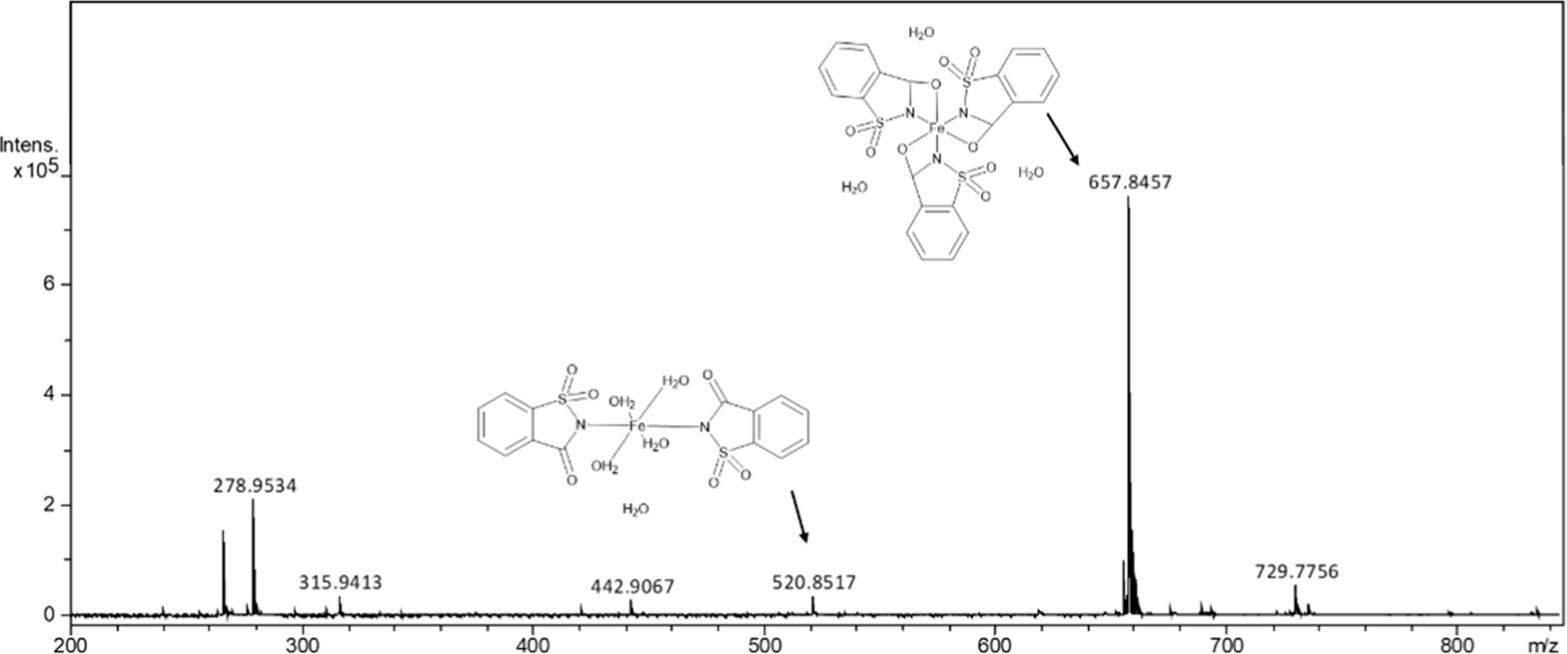
Mass spectra of the Fe–sac. The peaks corresponding to the complexes disaccharinate at *m*/*z* 520.8517 and trisaccharinate at *m*/*z* 657.8457.

The mass spectrum of the Co–sac complex (Figure 3) shows one prominent peak at *m*/*z* 663 corresponding to the formation of the complex with three saccharine ligands [Co­(C_7_H_4_SO_3_N)_3_]·3­(H_2_O) and the formation of the two saccharine ligands form [Co­(C_7_H_4_SO_3_N)_2_(H_2_O)_4_]·(H_2_O) at *m*/*z* 526.

**Figure 3:**
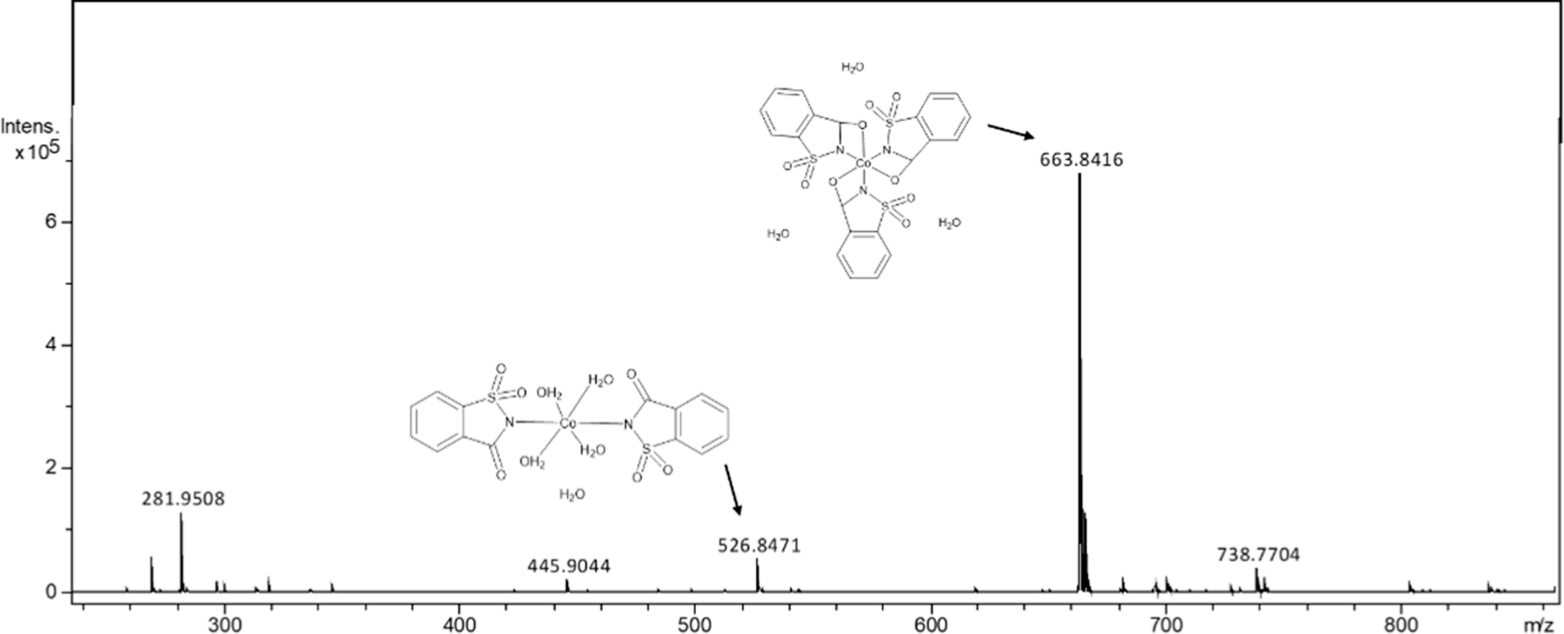
Mass spectra of Co–sac. The peaks corresponding to the complexes disaccharinate at *m*/*z* 526.8471 and trisaccharinate at *m*/*z* 663.8416.

The Ni–sac spectrum (Figure 4) shows *m*/*z* peaks with a distribution similar to that of Co–sac peaks, suggesting the formation of the trisaccharinate form [Ni­(C_7_H_4_SO_3_N)_3_]·3­(H_2_O) at *m*/*z* 663 and the formation of the disaccharinate form [Ni­(C_7_H_4_SO_3_N)_2_(H_2_O)_2_]·(H_2_O) at *m*/*z* 526.

**Figure 4:**
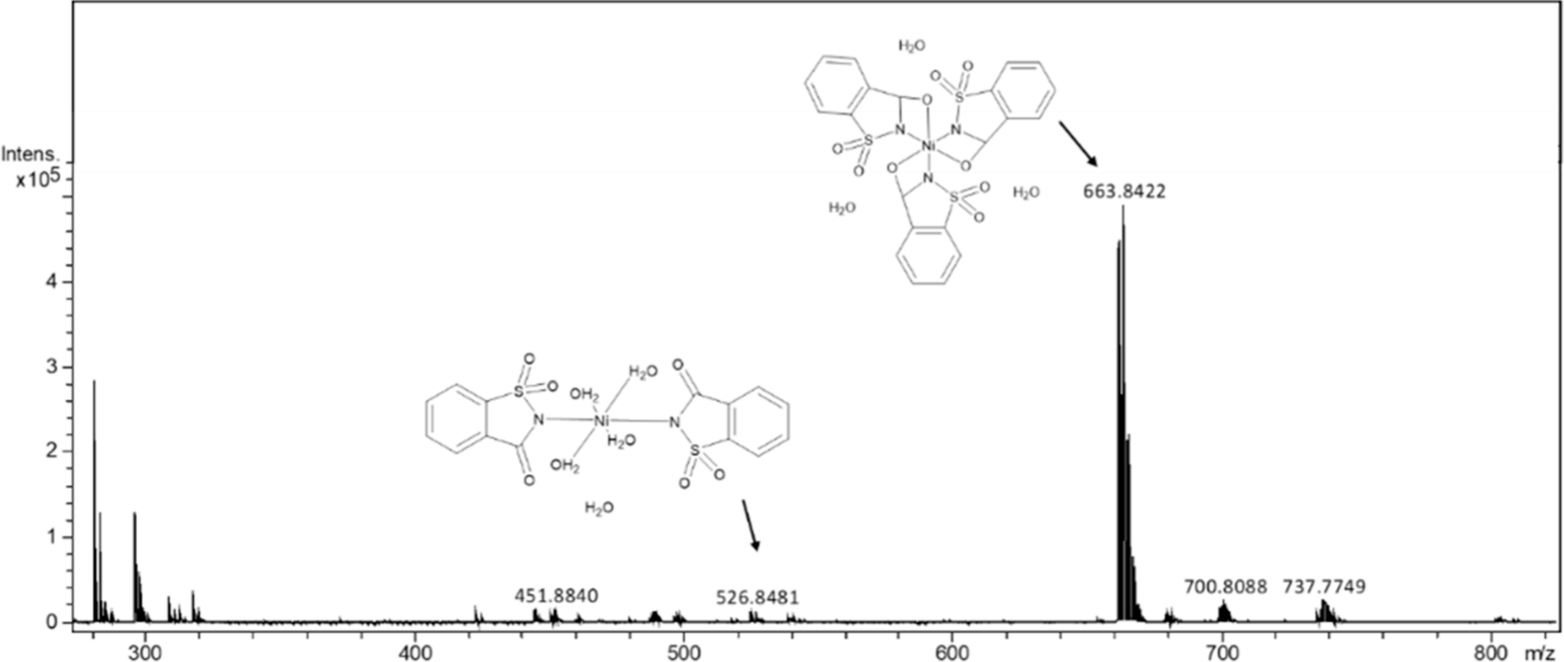
Mass spectra of Ni–sac. The peaks corresponding to the complexes disaccharinate at *m*/*z* 526.8481 and trisaccharinate at *m*/*z* 663.8422.

### 3.6 Electronic Spectra (UV–Vis)

The electronic spectra of the metal–saccharin complexes in solutions are shown in Figure S2. All solutions were prepared in methanol and evaluated in the 200 to 900 nm region. Molar absorptivity values (ε/L·mol^–1^·cm^–1^) for the characteristic wavelengths (λ) were calculated for the three complexes and are shown in Table 3.

**Table 3:** Data Extracted from the Electronic Spectra (Figure S2) and the Corresponding Assignments for the Transition Occurring in the Metal–Saccharine Complexes

complexes	λ (nm)	ε (L·mol^–1^·cm^–1^)
Na–sac	268	1.8 × 10^2^
Co–sac	268	3.2 × 10^2^
	511	1.2 × 10^3^
Fe–sac	268	2.7 × 10^2^
Ni–sac	268	2.9 × 10^2^
	400	0.7 × 10^2^
	668	2.7 × 10^2^
	735	2.8 × 10^2^

Figure S2a shows a high-intensity band in 268 nm (ε = 1.8 × 10^2^ L·mol^–1^·cm^–1^) referring to the sodium saccharin-free ligand. In the electronic spectrum of Figure S2, the same band is observed for metal–saccharin complexes, confirming the presence of saccharin coordinated to the metal ion, a high-intensity band found at 268 nm (ε = 3.2 × 10^2^ L mol^–1^ cm^–1^). The band refers to saccharin’s intraligand L → L* (IL) transitions.

In Figure S2b, the main band at 511 nm (ε = 1.2 × 10^3^ L·mol^–1^·cm^–1^) coincides with the ligand field transition ^4^T_1g_(P) → ^4^T_1g_ observed at 487 nm (Figure 5) for the octahedral [Co­(sac)_2_]^2+^ complex, with 10 Dq = 9200 cm^–1^. The intensity is too large for a Laporte forbidden transition, indicating the absence of an inversion center in the complex.^45^

**Figure 5:**
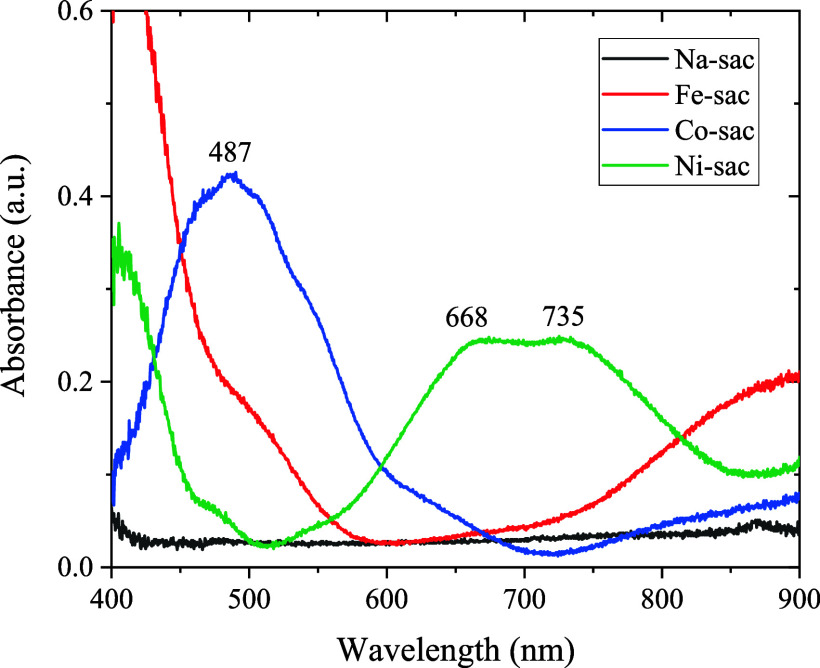
Diffuse reflectance electronic spectra of metal–saccharin complexes in solid form.

In the Ni–sac complex, two well-defined bands were observed at 268 and 668–735 nm (Figure S2d), with molar absorptivity of 2.9 × 10^2^ and 0.7 × 10^2^ L·mol^–1^·cm^–1^. According to the literature, the first band refers to the intraligand transition of saccharin. In contrast, the low-energy band at 735 nm (Figure 5) coincides with the ligand field transition ^3^T_1g_ → ^3^A_2g_ observed at 724 nm for the octahedral [Ni­(H_2_O)_6_]^2+^ complex, corresponding with 10 Dq = 8500 cm^–1^. The intensity is too large for a Laporte forbidden transition, indicating the absence of an inversion center in the complex. The low-energy component at 668 nm is also observed in the octahedral [Ni­(H_2_O)_6_]^2+^ complex and is ascribed to spin–orbit coupling.^25,45^

The Fe–sac complex has an intraligand band at 268 nm (Figure S2c; ε = 2.7 × 10^2^ L·mol^–1^·cm^–1^) and a near shoulder at 349 nm (Figure 5), attributed to the ligand saccharin. The low-energy band at 900 nm is also observed in the complex [Fe­(H_2_O)_6_]^2+^; which corresponds to the ligand field transition ^5^Eg → ^5^T_2g_; with a Dq value of 10,000 cm^–1^.^27^ This band is part of the Jahn–Teller splitting characteristic of high-spin Fe­(II) complexes, with the second band only observed in the near-infrared region, around 1,100 nm.^45^

### 3.7 Single-Crystal X-ray diffraction

Single crystals of the Fe–, Co–, and Ni–sac complexes were successfully obtained through the slow evaporation of a methanol and diethyl ether mixture. Crystallographic parameters, data collection details, and structural refinement are compiled in Table 4. Although related complexes have been previously reported in the literature,^46^ the present structural elucidation confirms the formation of the expected coordination environment, supporting the results obtained from the characterization techniques in the solid state.

**Table 4:** Crystal Data and Data Collection, and Refinements of Complexes of Metal–Saccharine

	Fe–sac	Co–sac	Ni–sac
empirical formula	C_14_H_20_FeN_2_O_12_S_2_	C_14_H_20_CoN_2_O_12_S_2_	C_14_H_20_N_2_NiO_12_S_2_
formula weight	528.29	531.37	531.15
crystal system	monoclinic	monoclinic	monoclinic
space group	*P*2_1_/c	*P*2_1_/*a*	*P*2_1_/*c*
*T*/K	100 K	100 K	100 K
radiation, λ/Å	0.71073	0.71073	0.71073
unit cell (Å) dimensions *a*	7.8692(3)	7.6317(7)	7.8515(3)
*b*	16.0644(6)	16.0433(14)	16.0595(7)
*c*	7.6637(3)	7.8368(8)	7.5808(3)
α, (deg)	90	90	90
β	99.8030(10)	99.428(3)	99.8700(10)
γ	90	90	90
*V* (Å^3^)	954.65(6)	946.56(15)	941.72(7)
*Z*	2	2	2
calculated density (g cm^–3^)	1.838	1.848	1.873
absorption coefficient (mm^–1^)	1.080	1.199	1.325
*F* (000)	544	546	548
crystal size (mm)	0.30 × 0.18 × 0.11	0.25 × 0.19 × 0.14	0.46 × 0.40 × 0.31
theta range for data collection	2.54–30.59	2.63–28.33	2.63–30.07
index ranges	–11 ≤ *h* ≤ 11	–10 ≤ *h* ≤ 10	–11 ≤ *h* ≤ 11
	–22 ≤ *k* ≤ 22	–19 ≤ *k* ≤ 21	–22 ≤ *k* ≤ 22
	–10 ≤ *l* ≤ 7	–9 ≤ *l* ≤ 10	–10 ≤ *l* ≤ 8
reflections collected/unique	26,797/2923 [*R*(int) = 0.0228]	13,343/2326 [*R*(int) = 0.0381]	19,914/2747 [*R*(int) = 0.0221]
completeness to theta max	99.7%	99.8%	99.7%
absorption correction	multiscan	multiscan	multiscan
max. and min transmission	0.7461 and 0.6637	0.7457 and 0.4207	0.6843 and 0.5810
refinement method	full-matrix least-squares on *F* ^2^	full-matrix least-squares on *F* ^2^	full-matrix least-squares on *F* ^2^
data/restraints/parameters	2923/0/160	2361/3/142	2747/3/142
goodness-of-fit on *F* ^2^	1.116	0.904	0.815
final *R* indices [*I* > 2σ(*I*)]	*R*1 = 0.0224, w*R*2 = 0.0643	*R*1 = 0.0321, w*R*2 = 0.0886	*R*1 = 0.0218, w*R*2 = 0.0560
*R* indices	*R*1 = 0.0250, w*R*2 = 0.0658	*R*1 = 0.0323, w*R*2 = 0.0889	*R*1 = 0.0222, w*R*2 = 0.0564
largest diff. peak and hole (e Å^–3^)	0.545 and −0.420	0.621 and −0.764	0.478 and −0.472

Figure 6–8 display the molecular structures of the complexes. Each metal center (Fe^2+^, Co^2+^, or Ni^2+^) is coordinated by two deprotonated saccharinate ligands in a monodentate form via a nitrogen atom and four water molecules. The saccharin ligands adopt a trans arrangement, while the coordinated water molecules occupy cis and trans positions within the octahedral coordination geometry. The bond lengths and angles for each complex are presented in Table S1, further confirming the metal–saccharin’s expected geometry and coordination behavior.

**Figure 6:**
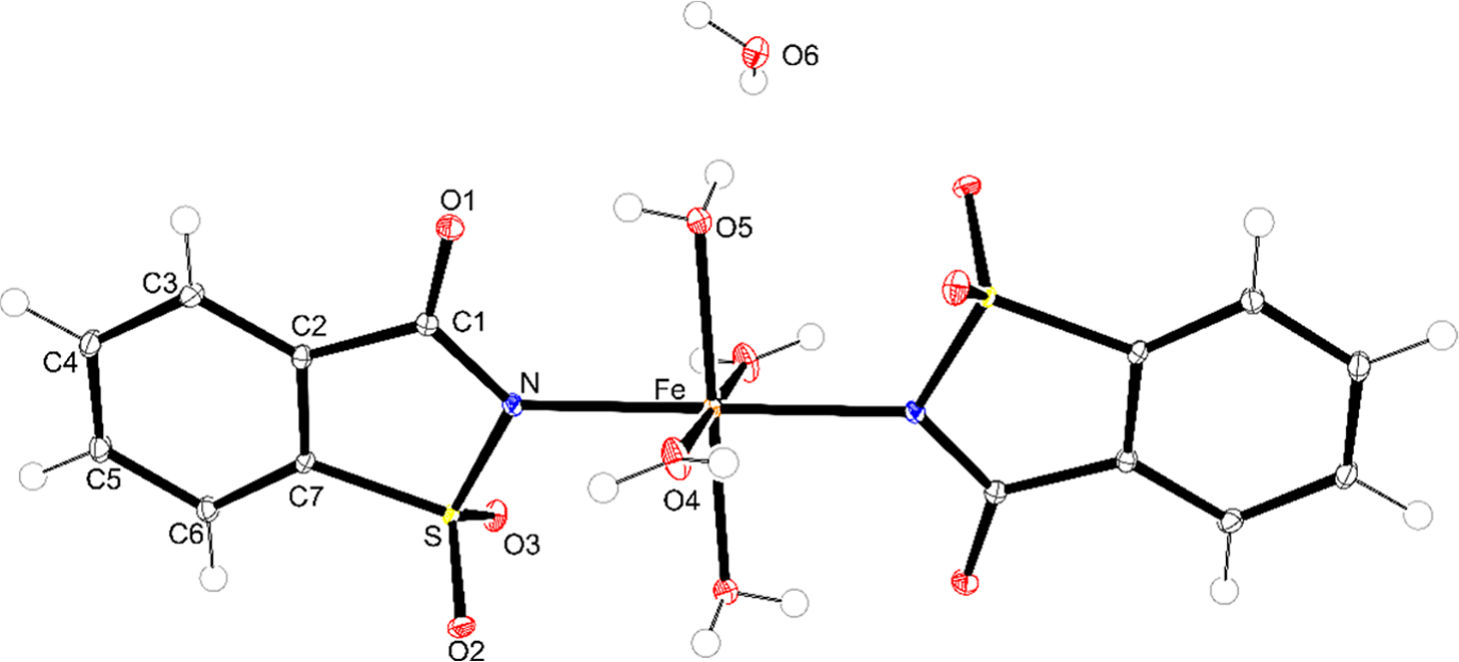
Representation of the tetraaquabis­(1,1,3-trioxo-3*H*-1,2-benzoisothiazol-2-yl)-iron­(II) complex (ellipsoids with 50% probability).

**7:**
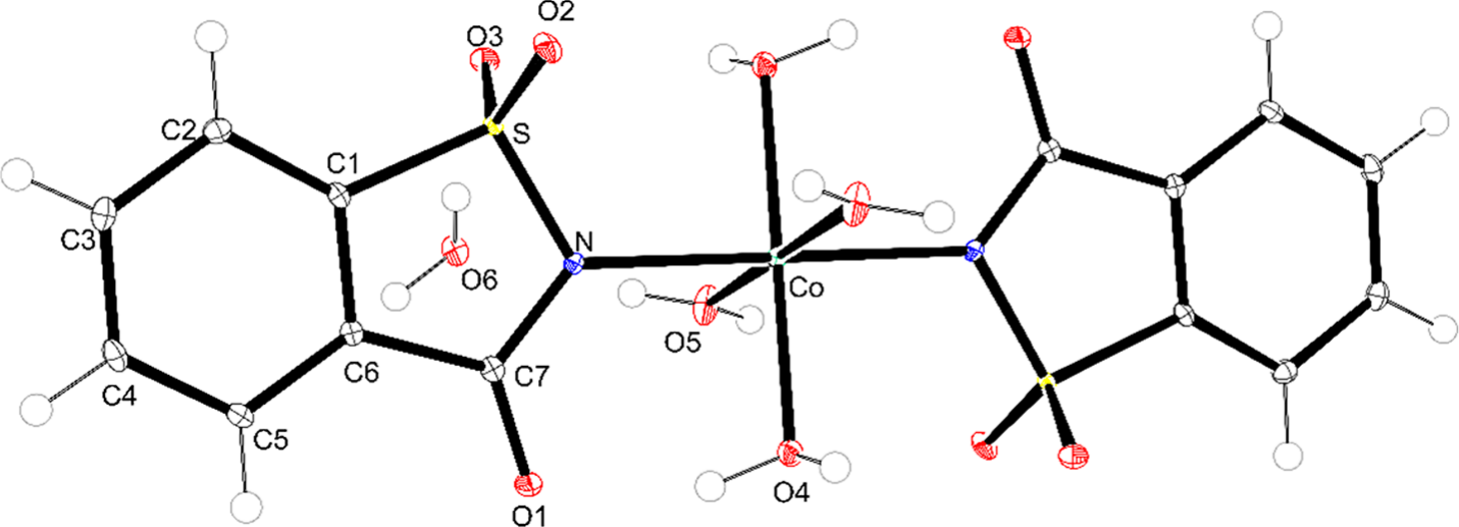
Representation of the tetraaquabis­(1,1,3-trioxo-3*H*-1,2-benzoisothiazol-2-yl)-cobalt­(II) complex (ellipsoids with 50% probability).

**Figure 8:**
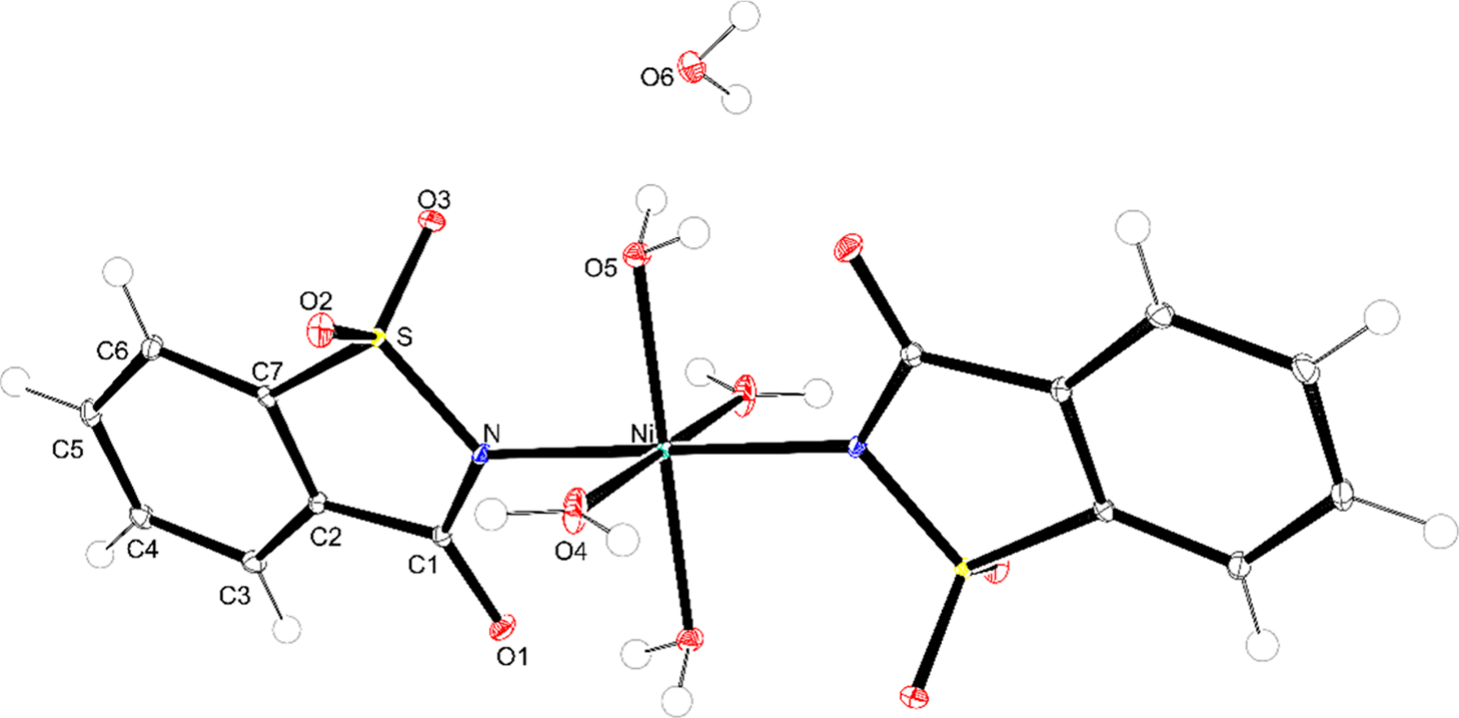
Representation of the tetraaquabis­(1,1,3-trioxo-3*H*-1,2-benzoisothiazol-2-yl)-nickel­(II) complex (ellipsoids with 50% probability).

Comparing with the literature, the complexes reported by Haider et al. (1983)^46^ with formula [M­(sac)_2_(H_2_O)_4_]·2H_2_O (M = Fe, Co, Ni) reveal that the complexes described here share several crystallographic features, including monoclinic symmetry and similar unit cell dimensions. For the Co–sac complex, the space group differs from those obtained in this work as they crystallized in the *P*2_1_/*a* space group instead of *P*2_1_/*c*.

### 3.8 Colorimetry Measurements

The colorimetric measurements (CIEL*a*b*) were performed in the powder complexes. In the coordinates L*a*b*, the coordinate L* indicates the luminosity varying from black to white, being assigned a scale of 0 for black and 100 for white, a* portrays the a* axis represents the color axis that ranges from red (+) to green (−); and the b* axis ranges from blue (−) to yellow (+).^47^

Sodium saccharin tended more toward white with high luminosity. Co–sac presented the highest value of a* and tended toward the red, while the Fe–sac presented the highest value of b* and tended toward the yellow. The Ni–sac presented the lowest value of a* and tended toward the green color. The values of L*a*b* found for the complex are expressed in Table 5.

**Table 5:**
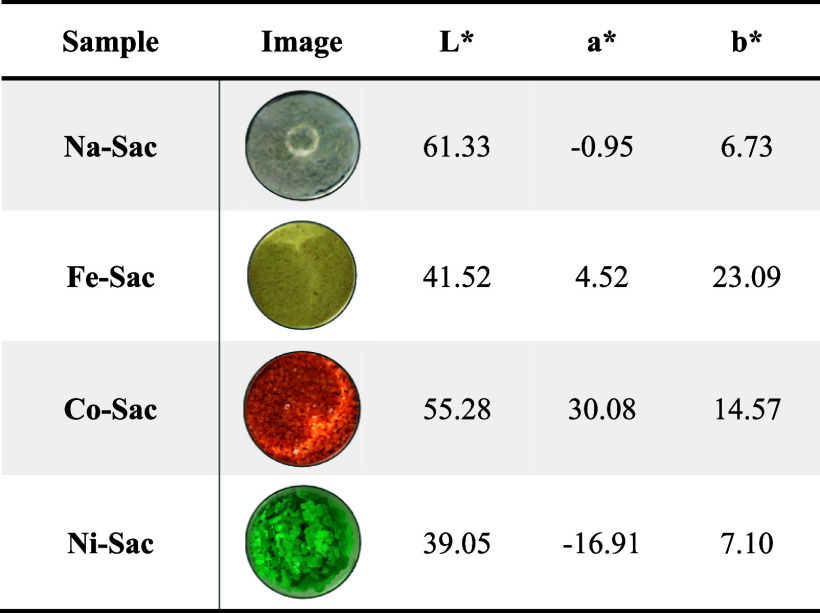
Colorimetric Parameters Obtained for Metal–Saccharin Complexes According to the CIEL*a*b* Color Space

### 3.9 Antimicrobial Activities

Table 6 shows the inhibition zones (mm) produced by the acrylic paint (AP) or respiratory mask (RM) samples containing the complexes (Co–sac and Ni–sac) against the microorganisms tested in the disk diffusion assay. The dispersion of the Fe–sac sample in the studied media (AP and RM) was incompatible.

**Table 6:** Diameters of the Inhibition Zones Formed by the Sample Dispersed in Acrylic Paste (AP or a Respiratory Mask (RM) Coated with Paint-Containing Complexes at Concentrations of 10% and 20% (w/w), Tested against Microorganisms^a^

	S. typhimurium	S. aureus		E. coli	L. monocytogenes
sample	inhibition halo AP (mm)	inhibition halo AP (mm)	inhibition halo RM (mm)	inhibition halo RM (mm)	inhibition halo RM (mm)
Co–sac 5%	11.20 ± 0.28	8.00 ± 0.80	6.98 ± 1.49	11.65 ± 2.25	---
Co–sac 10%	10.65 ± 0.19	14.03 ± 0.39	9.15 ± 0.82	11.58 ± 0.36	8.78 ± 2.34
Ni–sac 5%	9.15 ± 0.18	---	---	---	---

a “---” shows bacterial resistance (no inhibition halo).

Notably, the Ni–sac 5% solution exhibited antibacterial activity exclusively against *Salmonella typhimurium*, producing an inhibition halo of 9.15 ± 0.18 mm when incorporated into the acrylic paste. Compared to the same strain and material (AP), the sample that exhibited the greatest inhibition halo had the lowest concentration of Co–sac, 5%, followed by Co–sac 10%. However, there was no significant variation between the values of the cobalt samples, as shown in Table 6.

In contrast, for *S. aureus* strains tested in AP, Co–sac 5% and Co–sac 10% exhibited opposing activity and distinct behavior, with inhibition zone values of 8.00 ± 0.80 and 14.03 ± 0.39 mm, respectively. Notably, the Co–sac 10% pigment exhibits a greater antimicrobial susceptibility against *S. aureus*. This result reinforces the potential of cobalt-based pigments, particularly at higher concentrations, in inhibiting the growth of Gram-positive bacteria. Images of the antibacterial experiments are shown in Figure 9.

**Figure 9:**
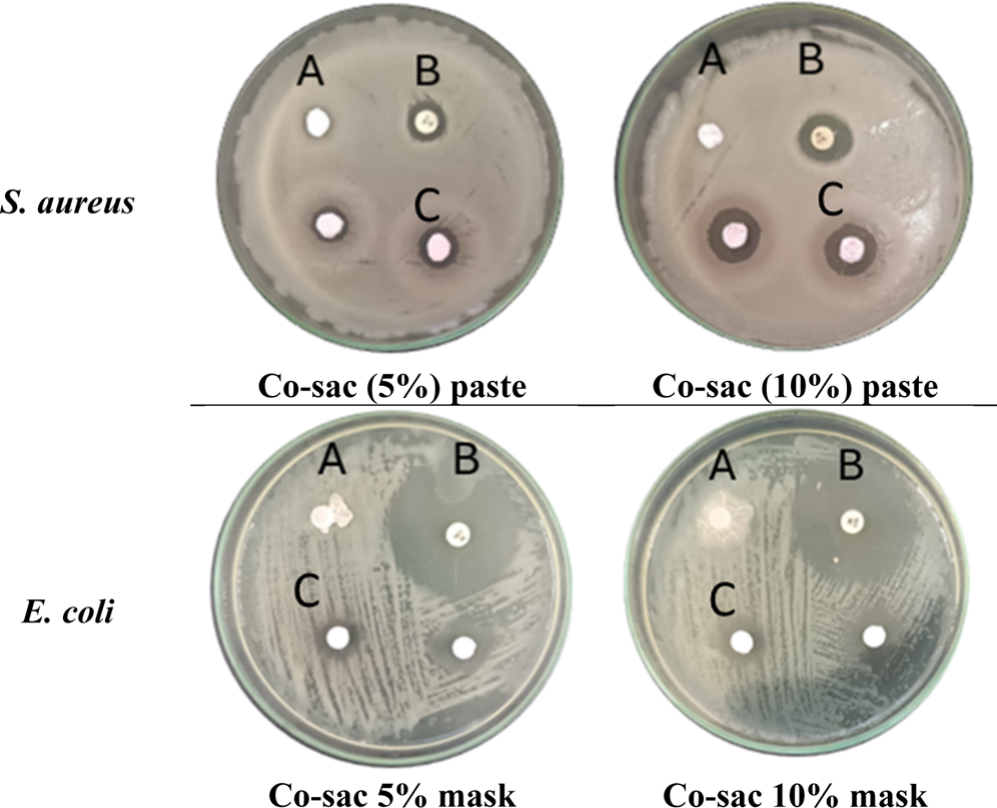
Antibacterial tests of Co–saccharine complex dispersed in ink on the mask using the diffusion disk method. Legend: “A” represents the pure mask or the pure acrylic paste; “B” corresponds to the commercial antibiotic tetracycline; and “C” denotes the samples of the impregnated complex (M–sac—paste or mask), tested in duplicate.

The Co–sac 5% complex incorporated in the respiratory mask (RM) did not show antibacterial activity against *L. monocytogenes*, and low efficacy against *S. aureus* (6.98 ± 1.49 mm), but possessed superior inhibition activity against the *E. coli* (11.65 ± 2.25 mm) strain.

In contrast, the Co-10% sample (in either AP or RM) displayed a broader antimicrobial spectrum, effectively inhibiting all microorganisms tested in this study. Inhibition zones generally exceeded 9 mm for most Gram-positive and Gram-negative strains. Specifically, for the RM sample, the inhibition diameters were 11.58 ± 0.36 mm for *E. coli*, 9.15 ± 0.82 mm for *S. aureus*, and 8.78 ± 2.34 mm for *L. monocytogenes*, respectively.

Additionally, acrylic paste’s best antibacterial activity was observed against Gram-positive bacteria, whereas the strongest effect was against Gram-negative strains in the respiratory mask.

Figure 10a illustrates the inhibition halos of the complexes (incorporated into the RM mask) compared to those of the standard antibiotic for each respective strain. The relationship between the obtained diameter values determines the activity index (AI), which was calculated for all samples relative to the standard antimicrobial agents, tetracycline (Tcy) or norfloxacin (Nor). The lowest antibacterial activity index was observed in Co–Sac 5% (RM) against *E. coli* (AI = 0.37) and *S. aureus* (AI = 0.43) as well as in Co–Sac 10% (RM) against *S. aureus* (AI = 0.39). Similar activity index was seen by Co–sac 10% (RM) against *E. coli* (AI = 0.53) and Co–sac 5% (AP) against *S. aureus* (AI = 0.54).

**Figure 10:**
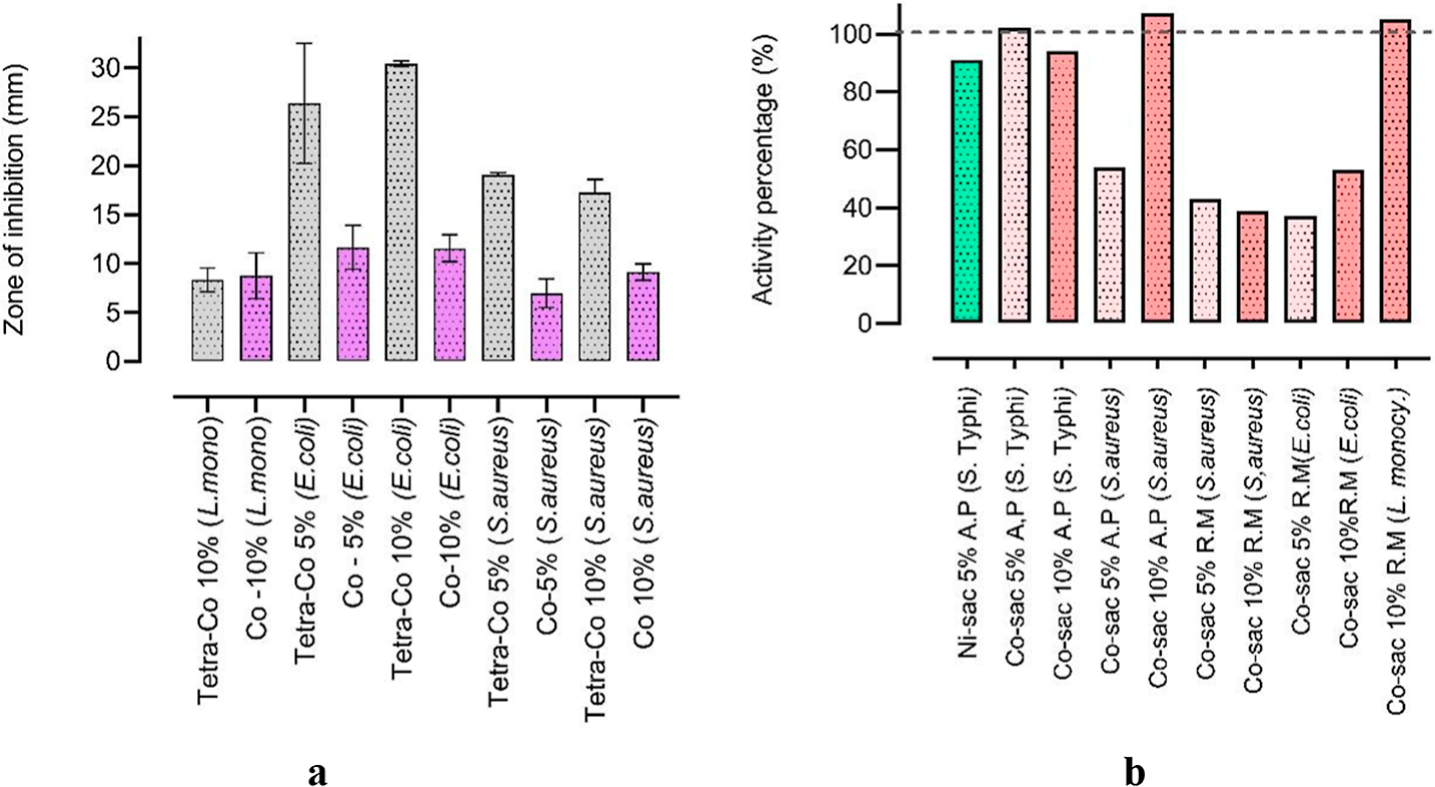
(a) Diffusion halo results of the complex in the mask (RM) compared to the standard antibiotic (tetracycline, Tcy). (b) Percentage activity (%) of all samples in different microorganisms.

Furthermore, the promising results obtained against *S. typhimurium* gave an activity index of 1.02 (Co–Sac 5% AP) and a high inhibition index against *L. monocytogenes*, with AI = 1.05 (Co–Sac 10% RM). These inhibition zones correspond to 102% and 105% (Figure 10b) of the values observed with the two reference antibiotics (Tcy, Nor), highlighting the tested samples’ strong antimicrobial potential. All obtained values for the activity index were converted to activity percentages and are presented in Figure 10b.

Thus, as shown in Figure 10b, the percentage of activity for the samples in the mask was lower compared to that of the same compounds incorporated into the acrylic paste, except for the Co–sac 10% sample against *L. monocytogenes*, which showed 102% antibacterial activity. Notably, although the samples exhibited a good inhibition zone (Table 6), their activity percentage was lower than that of a standard antibiotic. This behavior can be attributed to the fabric’s incomplete absorption of the paint containing the M–sac complex. Further tests will be conducted soon to enhance the pharmacopeia information on these complexes in their antimicrobial activity.

The disk diffusion test with complexes dispersed in acrylic paste demonstrated that metal–saccharin complexes retain their antimicrobial properties even when embedded in another material. This finding suggests their potential use as antibacterial pigments for surface coatings.

Minimum inhibitory concentration (MIC) assays were performed for M–sac complexes (M–sac: Fe–sac, Co–sac, and Ni–sac) and the free ligand saccharin provided a deeper understanding of the antimicrobial potential of these compounds in solution. Two species of pathogenic microorganisms were selected for this test: the Gram-negative bacterial strain *Salmonella* enterica *Typhimurium* (ATCC 0280) and the fungus strain *C. albicans* (ATCC 10231).

Gram-negative bacterial strains are more resistant to the action of compounds, primarily due to the presence of an outer membrane that acts as a barrier, preventing them from entering the cellular environment. Invasive infections caused by Candida strains continue to be a significant cause of morbidity and mortality, and with the limited number of antifungal agents available, resistance to these drugs is only increasing. *C. albicans* is among the leading causes of invasive candidiasis globally.^48–50^

The MIC (μg μL^–1^) values for each complex are outlined below. The concentrations assessed were 1000–62.5 μg μL^–1^. The free ligand did not display antibacterial or antifungal activity in the concentration range studied in this work. The Co–sac complex exhibited the best antimicrobial activity among all of the compounds studied. The compound exhibited a minimum inhibitory concentration (MIC) of 62.5 μg mL^–1^against *S. Typhimurium* strains, with biocidal activity observed at 125 μg μL^–1^.

Notably, for the *C. albicans* strain, a surprising result was recorded, with a MIC below 62.5 μg μL^–1^ and a fungicidal effect at the same concentration. The Ni–sac complex showed better inhibitory activity for yeast (MIC = 250 μg mL^–1^) than the bacteria strains tested (MIC = 500 μg mL^–1^). It exhibited bacteriostatic activity, while yeast fungicidal activity was observed at a concentration of 500 μg mL^–1^. The Fe–sac complex showed no measurable MIC against Gram-negative *S. Typhimurium* at different concentrations. In contrast, the compound exhibited an MIC of 500 μg mL^–1^ against *C. albicans* and the fungicidal effect (MBC) observed at 1000 μg mL^–1^.

By comparing the results of this work, it was observed that the complexes that obtained the best MIC values for bacteria and fungi were Co–sac and Ni–sac. The compound Co–sac inhibited effective pathogenic activity against all microorganisms and was demonstrated to be a strong bioinorganic complex with the potential to act as an antibacterial and antifungal agent.

In the study reported by Kumar et al. (2020) for the complex series with the formula [M­(dppz)_2_(sac)­(H_2_O)]­ClO_4_ (where the ligand dppz: dipyridophenazine) also containing the ligand saccharin and varying the metal center (M: Co­(II), Ni­(II), and Cu­(II)), the complexes were studied in the MIC test.^30^ Thus, comparing the results of the literature with the Co–sac compound synthesized in this study (MIC = 62.5 mg. ml^–1^, for *S. Typhimurium*), it is observed that the complexes [M­(dppz)_2_(sac)­(H_2_O)] ClO_4_, with metallic center of Co­(II), presented a MIC of 64 μg μL ^–1^ for strain *E. coli* and Ni­(II) exhibited an MIC of 64 μg μL ^–1^ for *E. coli* strain. By comparison, the complexes synthesized and tested in this study had the same or better chemistry capacity to inhibit pathogenic microorganisms at lower concentrations.

Finally, the results presented by Co–sac complexes of this study (MIC = 62.5 mg. ml^–1^, for *S. Typhimurium*) are so similar to former data analyzed by Ravoof et al. (2004) for the complex [Cu­(NNS′)­(sac)] (ligand NNS′: *S*-methyl-β-*N*-(6-methylpyrid-2yl)­methylenedithiocarbazate), which exhibited an MIC of 62.5 μg/μL for the *S. Typhimurium* and *C. albicans*, respectively.^51^

## 4 Conclusions

Iron­(II), cobalt­(II), and nickel­(II) complexes containing a saccharine ligand were successfully synthesized and characterized by molar conductivity, thermogravimetry, FTIR, mass spectroscopy, UV–vis, 1H NMR, single-crystal X-ray diffraction, and colorimetry. The results confirmed the saccharin ligand’s ability to coordinate with metal centers, forming stable complexes with distinct spectroscopic profiles.

The compounds were evenly dispersed in acrylic paste and fabric paint on the mask and maintained their antimicrobial activity. The Fe–saccharine complex does not present an inhibition capacity against any bacteria during its growth; Ni–saccharine showed moderate inhibition activity. On the other hand, Co–saccharine demonstrated better microbiological properties for inhibiting several pathogenic microorganisms. Hence, when dispersed in acrylic paste, Co–sac (5%) had better antimicrobial activity against *S. Typhimurium*; however, Co–sac (10%) had the best antimicrobial activity against *S. aureus*. When dispersed on the mask, Co–sac (5%) demonstrated to be the better inhibitor against *S. aureus*, and for *E. coli*, Co–saccharine at 10% concentration demonstrated the best antimicrobial capacity. Moreover, for the yeast *L. monocytogenes*, only Co–sac (10%) showed strong effectiveness.

These results highlight the potential of metal–saccharinate complexes as multifunctional antimicrobial agents. Their compatibility with coating materials makes them promising candidates for use as protective surfaces and adsorbents embedded in textiles or fabric in contexts where microbial control is essential, such as healthcare environments and personal protective equipment.

## Supplementary Material



## Notes

Complementary information, such as TG/DTG curves; electronic spectra in the UV–vis region; NMR spectrum; and a table containing bond distance and angle related to single-crystal X-ray diffraction data (PDF)
