# Supplementary material for: Synthesis of Metal–Saccharin Complexes as an Antimicrobial Inorganic Pigment for Surfaces
Source: ACS Omega. 2025 Nov 12;10(46):56130–42. doi: 10.1021/acsomega.5c07778 (PMC12658693; doi:10.1021/acsomega.5c07778)
Supplement: Supplementary file 1 [file ao5c07778_si_001.pdf]

SUPPORTING INFORMATION

Synthesis of metal-saccharin complexes as an antimicrobial inorganic pigment for surfaces

Camila Acorone Soares[1], Patrícia Appelt[1], Weslei Domingos da Silva[2], Mário Antônio Alves da Cunha[2], Tulio Chavez-Gil[3], Henrique E. Toma[4], Davi F. Back[5], Fauze Jacó Anaissi[1,*]

[1] Department of Chemistry, Campus Cedeteg, Universidade Estadual do Centro-Oeste, 85040-080, Guarapuava, Brazil

[2] Department of Chemistry, Campus Pato Branco, Universidade Tecnológica Federal do Paraná, 85503-390, Pato Branco, Brazil

[3] Laboratory of Advanced Biofuels and Biomaterials, Department of Natural Sciences, Coppin State University, 2500 W North Ave, Baltimore, Maryland, U.S.A

[4] Department of Chemistry, University of São Paulo (USP), Avenue Professor Lineu Prestes, São Paulo 05508-000, São Paulo, Brazil

[5] Department of Chemistry, Universidade Tecnológica Federal de Santa Maria (UFSM), Avenue Roraima nº 1000, Santa Maria 97105-900, Rio Grande do Sul, Brazil

*E-mail: anaissi@unicentro.br

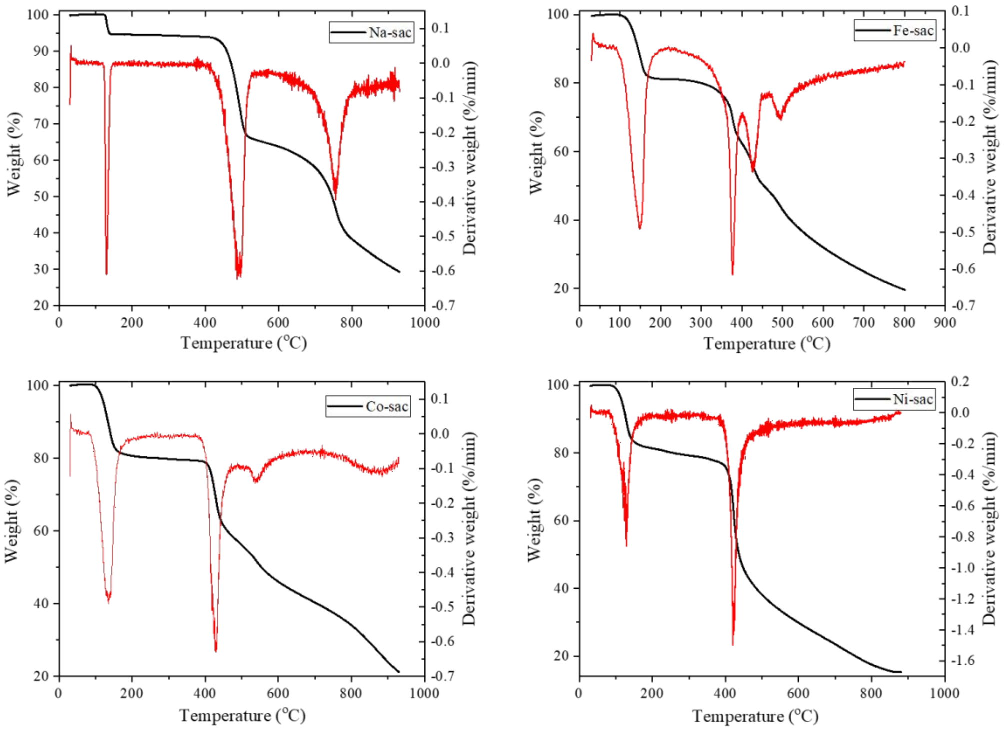

Figure S1. TG and dTG curves of metal-saccharinate complexes.

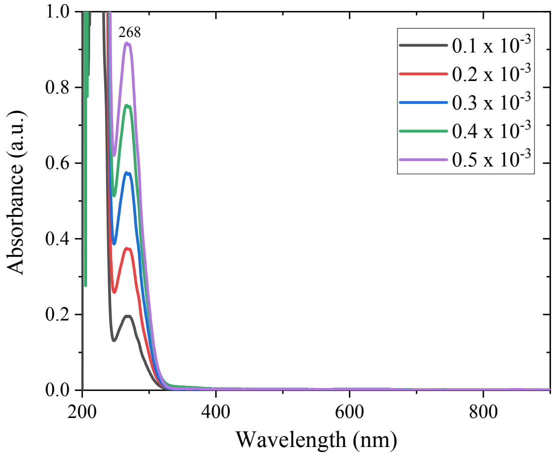

a) Na-sac

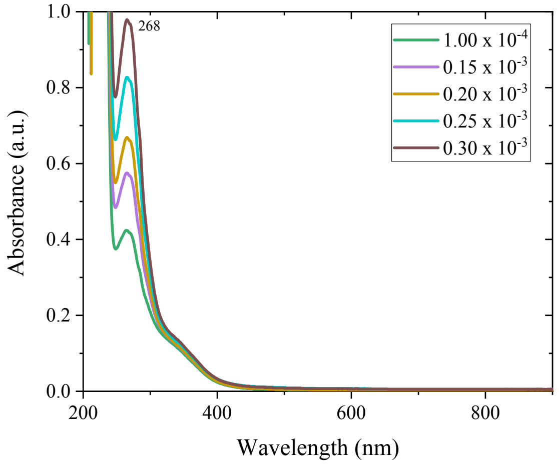

b) Fe-sac

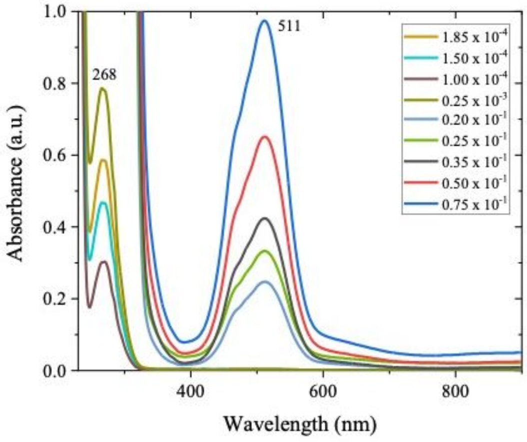

c) Co-sac

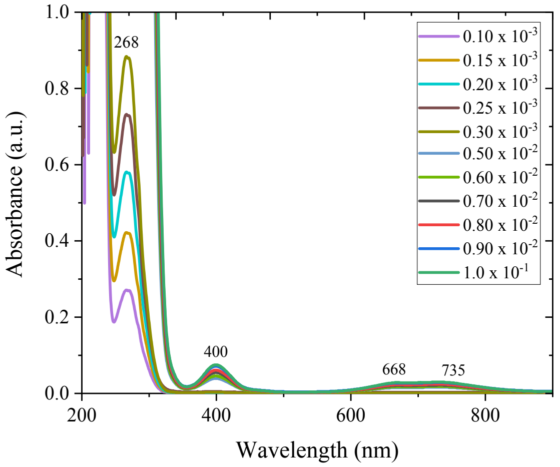

d) Ni-sac

Figure S2. Electronic spectra in the UV-Vis region for the metal complexes with saccharin as a ligand in methanol solution. (a) Na-sac; (b) Fe-sac; (c) Co-sac; (d) Ni-sac.

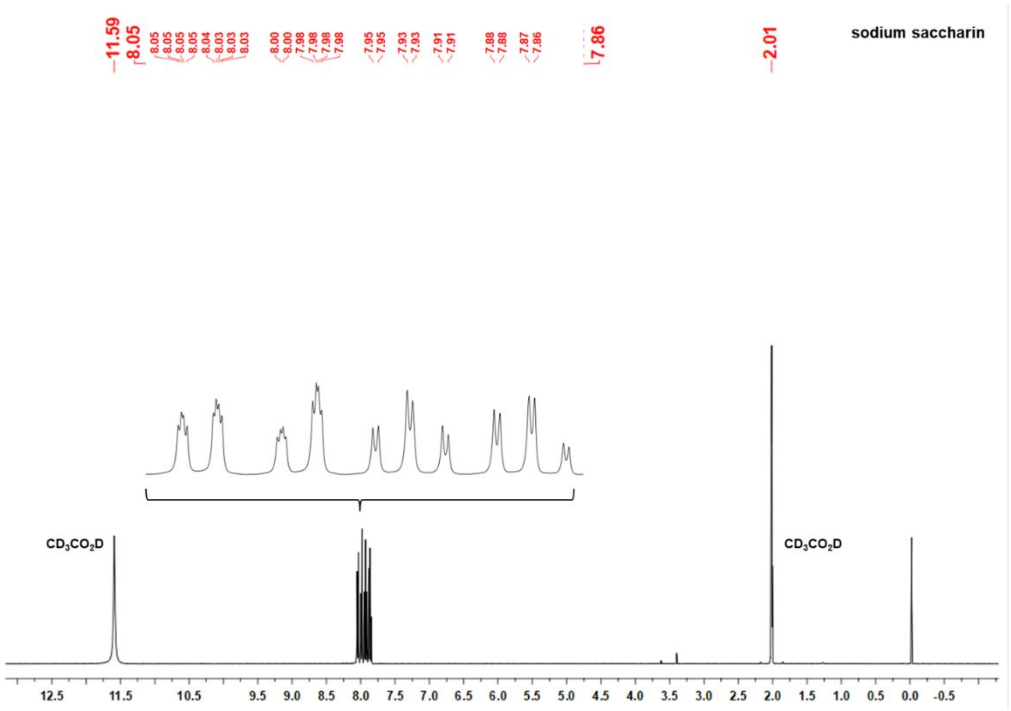

Figure S3.1 . NMR ^{1}H spectrum of Sodium saccharin (Sac-Na).

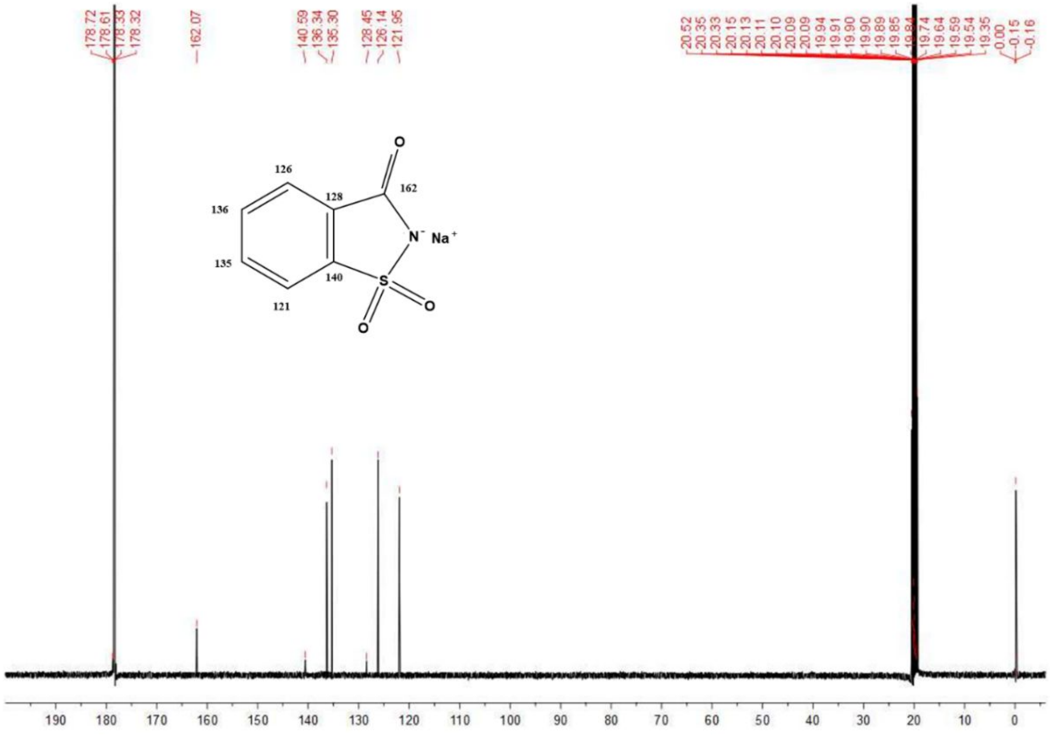

Figure S3.2 . NMR ^{13}C spectrum of Sodium saccharin (Sac-Na).

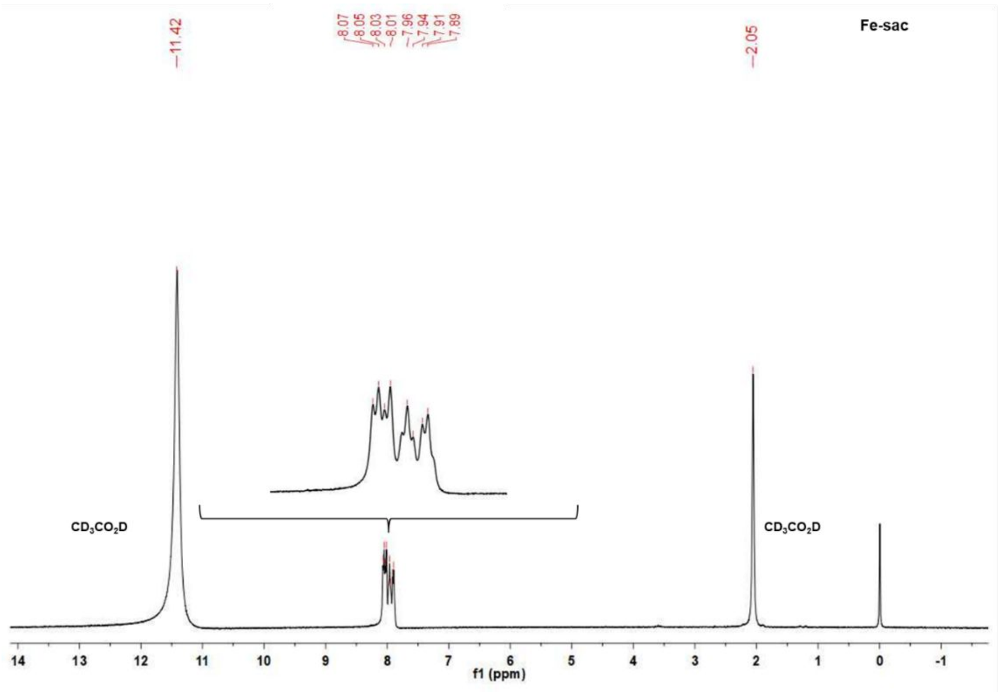

Figure S3.3 NMR ^{1}H spectrum of Fe-sac.

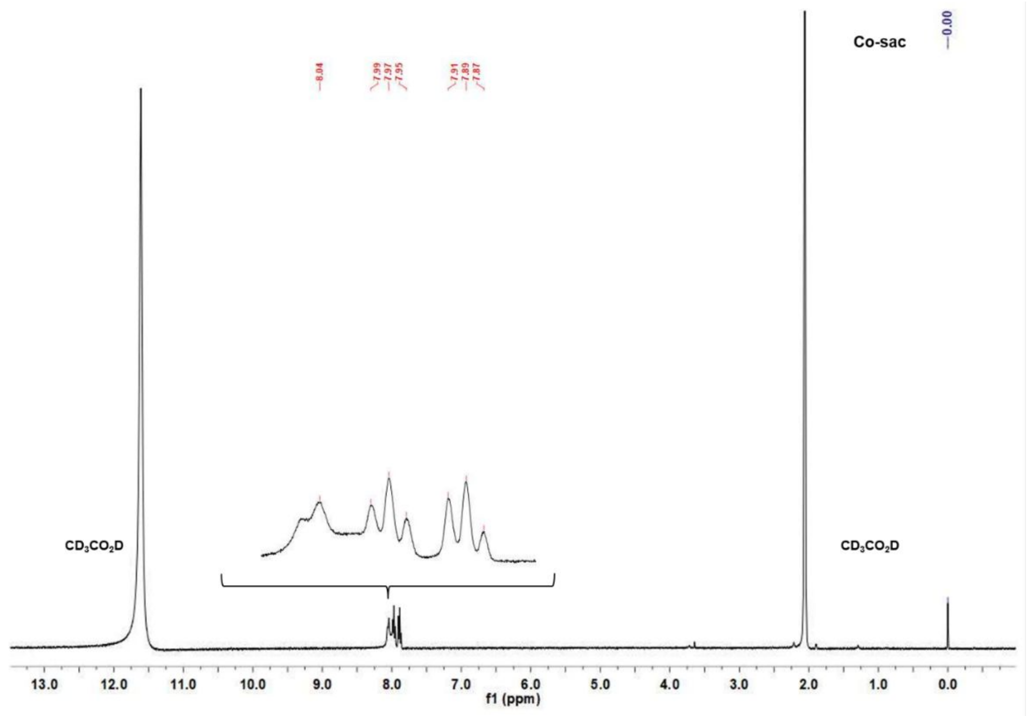

Figure S3.4 NMR ^{1}H spectrum of Co-sac

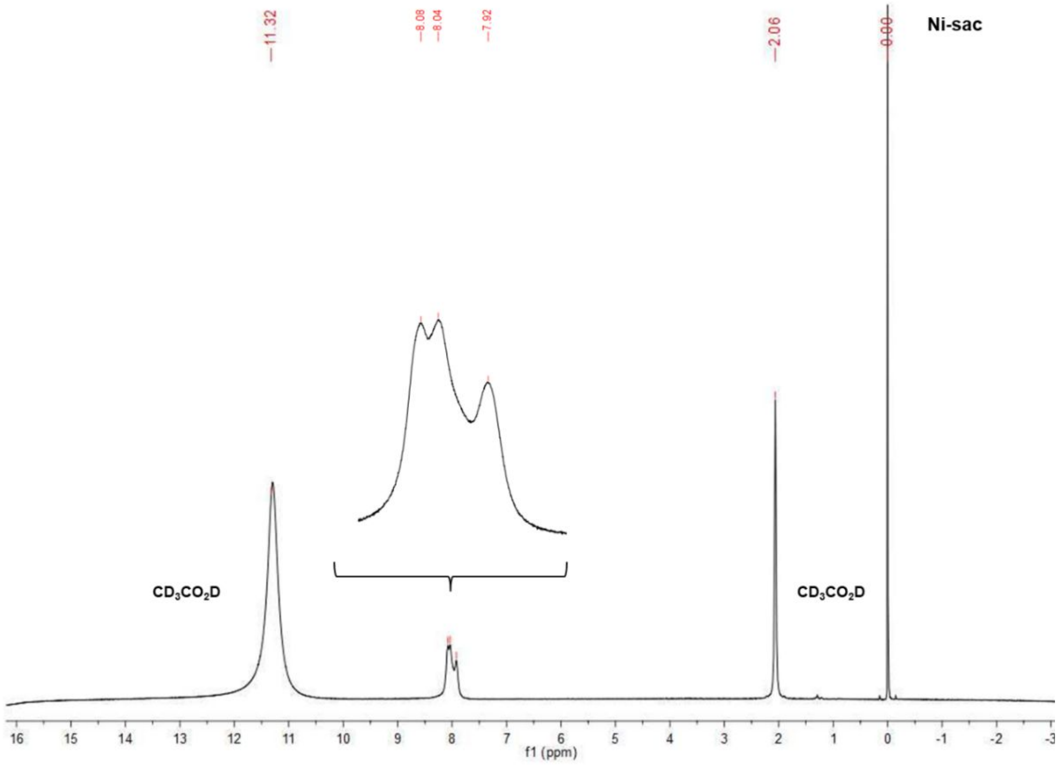

Figure S3.5 NMR ^{1}H spectrum of Ni-sac.

Table S1. The bonds lengths and angles values related of the complexes Fe-sac, Co-sac and Ni-sac.

Fe-sac		Co-sac		Ni-sac	
**Bond distance (Å)**					
Fe-O(5)	2.0994(9)	Co-O(4)	2.0648(18)	Ni-O(5)	2.0431(12)
Fe-O(4)	2.1527(9)	Co-O(5)	2.1147(18)	Ni-O(4)	2.0905(12)
Fe-N	2.2326(9)	Co-N	2.200(2)	Ni-N	2.1515(13)
S-O(3)	1.4424(9)	S-O(2)	1.4410(18)	S-O(2)	1.4407(12)
S-O(2)	1.4501(9)	S-O(3)	1.4511(19)	S-O(3)	1.4497(12)
S-N	1.6354(10)	S-N	1.638(2)	S-N	1.6415(14)
S-C(7)	1.7507(11)	S-C(1)	1.747(2)	S-C(7)	1.7479(16)
O(1)-C(1)	1.2465(14)	O(1)-C(7)	1.245(3)	O(1)-C(1)	1.2459(19)
N-C(1)	1.3608(14)	N-C(7)	1.360(3)	N-C(1)	1.365(2)
C(1)-C(2)	1.4990(15)	C(6)-C(1)	1.387(3)	C(1)-C(2)	1.499(2)
C(2)-C(3)	1.3880(15)	C(6)-C(5)	1.391(3)	C(2)-C(7)	1.387(2)
C(2)-C(7)	1.3883(15)	C(6)-C(7)	1.501(3)	C(2)-C(3)	1.390(2)
C(3)-C(4)	1.3986(16)	C(1)-C(2)	1.387(3)	C(3)-C(4)	1.399(2)
C(4)-C(5)	1.3935(16)	C(2)-C(3)	1.391(3)	C(4)-C(5)	1.396(2)
C(5)-C(6)	1.3972(15)	C(4)-C(5)	1.397(3)	C(5)-C(6)	1.395(2)
C(6)-C(7)	1.3838(15)	C(4)-C(3)	1.395(4)	C(6)-C(7)	1.385(2)
Angle (°)					
O(5)-Fe-O(4)	89.84(4)	O(4)-Co-O(5)	88.76(7)	O(5)-Ni-O(4)	88.73(5)
O(5)-Fe-N	86.71(3)	O(4)-Co-N	87.71(7)	O(5)-Ni-N	91.58(5)
O(4)-Fe-N	86.84(4)	O(5)-Co-N	86.70(7)	O(4)-Ni-N	92.18(5)
O(3)-S-O(2)	116.22(5)	O(2)-S-O(3)	116.39(11)	O(2)-S-O(3)	116.76(8)
O(3)-S-N	110.98(5)	O(2)-S-N	110.86(11)	O(2)-S-N	110.93(7)
O(2)-S-N	109.48(5)	O(3)-S-N	109.69(11)	O(3)-S-N	109.57(7)
O(3)-S-C(7)	111.21(5)	O(2)-S-C(1)	111.12(11)	O(2)-S-C(7)	111.08(7)
O(2)-S-C(7)	110.24(5)	O(3)-S-C(1)	109.97(11)	O(3)-S-C(7)	109.66(7)
N-S-C(7)	97.05(5)	N-S-C(1)	97.12(11)	N-S-C(7)	97.07(7)
C(1)-N-S	110.82(8)	C(7)-N-S	110.72(17)	C(1)-N-S	110.58(11)
C(1)-N-Fe	129.72(8)	C(7)-N-Co	129.54(16)	C(1)-N-Ni	129.74(11)
S-N-Fe	119.31(5)	S-N-Co	119.60(11)	S-N-Ni	119.53(7)
O(1)-C(1)-N	123.60(10)	C(1)-C(6)-C(5)	119.4(2)	O(1)-C(1)-N	123.81(15)
O(1)-C(1)-C(2)	123.03(10)	C(1)-C(6)-C(7)	111.1(2)	O(1)-C(1)-C(2)	122.89(14)
N-C(1)-C(2)	113.36(10)	C(5)-C(6)-C(7)	129.5(2)	N-C(1)-C(2)	113.30(13)
C(3)-C(2)-C(7)	119.41(10)	C(6)-C(1)-C(2)	123.9(2)	C(7)-C(2)-C(3)	119.36(14)
C(3)-C(2)-C(1)	129.49(10)	C(6)-C(1)-S	107.63(18)	C(7)-C(2)-C(1)	111.22(13)
C(7)-C(2)-C(1)	111.07(10)	C(2)-C(1)-S	128.52(19)	C(3)-C(2)-C(1)	129.39(14)
C(2)-C(3)-C(4)	118.04(11)	C(1)-C(2)-C(3)	116.3(2)	C(2)-C(3)-C(4)	118.00(15)
C(5)-C(4)-C(3)	121.38(11)	C(5)-C(4)-C(3)	121.4(2)	C(5)-C(4)-C(3)	121.39(15)
C(4)-C(5)-C(6)	121.06(10)	C(2)-C(3)-C(4)	121.1(2)	C(6)-C(5)-C(4)	121.01(15)
C(7)-C(6)-C(5)	116.18(10)	O(1)-C(7)-N	123.7(2)	C(7)-C(6)-C(5)	116.24(15)
C(6)-C(7)-C(2)	123.92(10)	O(1)-C(7)-C(6)	123.0(2)	C(6)-C(7)-C(2)	123.99(15)
C(6)-C(7)-S	128.50(9)	N-C(7)-C(6)	113.3(2)	C(6)-C(7)-S	128.33(12)
C(2)-C(7)-S	107.58(8)	C(6)-C(5)-C(4)	118.0(2)	C(2)-C(7)-S	107.68(11)
